# A Taxonomic Study of the *proclia* Group and Allied Species of *Aphytis* (Hymenoptera: Aphelinidae) from China, with Descriptions of Two New Species †

**DOI:** 10.3390/insects17090966

**Published:** 2026-09-19

**Authors:** Jingtao Xi, Zhigang Dong, Jian Huang, Zhuhong Wang

**Affiliations:** State Key Laboratory of Agricultural and Forestry Biosecurity, College of Plant Protection, Fujian Agriculture and Forestry University, Fuzhou 350002, China; 12302003026@fafu.edu.cn (J.X.); d18235546505@163.com (Z.D.); jhuang1234@126.com (J.H.)

**Keywords:** *Aphytis*, *proclia* group, allied species, new species, integrative taxonomy, molecular phylogeny, China

## Abstract

The genus *Aphytis* (Hymenoptera: Aphelinidae) comprises ectoparasitoids of scale insects and includes many species of biological control importance. The *proclia* group is a major lineage within this genus, with seven species recorded in China, along with two morphologically closely related species. Based on specimens collected from Fuzhou, Fujian Province, we identified four species: *A. hispanicus* (Mercet), *A. pinnaspidis* Rosen & DeBach, and two new species, *A. latarcuatus* Wang & Huang, sp. n. and *A. denticulatus* Wang & Huang, sp. n. The two new species are described and illustrated, and host associations and distribution records for all four species are summarized. A complete checklist and a morphological key for the 11 Chinese species of the *proclia* group and its morphologically similar species are provided. Molecular phylogenetic analyses based on partial COI and 28S rDNA sequences support the delimitation of the two new species and reveal a distinct lineage comprising *A. pinnaspidis* and the two new species which are excluded from the *proclia* group. This study presents the first molecular phylogenetic analysis of Chinese members of the *proclia* group and its allied species and highlights the value of integrating morphological and molecular evidence in species group-level taxonomy.

## Simple Summary

*Aphytis* species are tiny parasitic wasps that play a significant role in the biological control of scale insects, which are known pests in agriculture and forestry. The *proclia* group is one of the major species groups within this genus, with seven species and two allied species recorded in China. In this study, we collected four species of *Aphytis* from Fuzhou, Fujian Province, China. Two were identified as known species, *A. hispanicus* (Mercet) and *A. pinnaspidis* Rosen & DeBach, and two are described as new species: *A. latarcuatus* Wang & Huang, sp. n. and *A. denticulatus* Wang & Huang, sp. n. We provide detailed descriptions and illustrations for the two new species, along with a summary of hosts and distribution records for all collected species. Additionally, we present a complete checklist of the *proclia* group and its allied species known from China and provide a morphological key for their identification. To clarify their evolutionary relationships, we reconstructed phylogenetic trees using DNA sequences from two different genes. This study contributes to the taxonomy of Chinese *Aphytis* by integrating morphological and molecular evidence and provides a foundation for future research on the *proclia* group and its allied species.

## 1. Introduction

The genus *Aphytis* Howard (Hymenoptera: Aphelinidae) comprises predominantly ectoparasitoids of armored scale insects (Hemiptera: Diaspididae). Currently, 112 species of *Aphytis* have been recorded worldwide [1], of which 43 are known from China [2,3,4]. Many *Aphytis* species exert significant control over diaspidid pests and have been widely employed in biological control programs across various countries and regions [5,6,7].

In the taxonomic study of *Aphytis*, species with similar morphological features are traditionally grouped into informal assemblages termed “species groups”, which often reflect close phylogenetic relationships. Compere (1955) [8] first divided the genus into three species groups: *chrysomphali*, *proclia*, and *mytilaspidis*. Among these, seven species were initially assigned to the *proclia* group, but only three, *A. proclia* (Walker, 1839), *A. diaspidis* (Howard, 1881), and *A. maculicornis* (Masi, 1911), were considered valid at that time [8]. These species are characterized by black transverse stripes on both sides of the occiput and variable brown to dark brown markings on the body. Rosen and DeBach (1979) [9] revised the genus, recognizing 90 valid species and expanding the number of species groups to seven (with the *vittatus* group subsequently transferred to the genus *Paraphytis* Compere, 1925). Within this framework, eight additional species were assigned to the *proclia* group [9]: *A. paramaculicornis* DeBach & Rosen, 1976; *A. hispanicus* (Mercet, 1912); *A. comperei* DeBach & Rosen, 1976; *A. philippinensis* DeBach & Rosen, 1976; *A. vandenboschi* DeBach & Rosen, 1976; *A. confusus* DeBach & Rosen, 1976; *A. pinnaspidis* Rosen & DeBach, 1979; and *A. testaceus* Chumakova, 1961. Rosen and DeBach (1979) [9] redefined the diagnostic features of the *proclia* group as follows: body generally pale gray; six-segmented antennae in both sexes; occiput and genal sutures with distinct dark markings; body and appendages with dark brown maculations; thoracic sternum and apodeme conspicuously darkened; forewing setae uniform, with a distinct infuscate spot beneath the stigmal vein and at the junction of the submarginal and marginal veins; pupae usually bearing dark brown markings. Since the 1980s, only two additional species have been added to the *proclia* group [10,11]: *A. manii* Hayat, 1998 and *A. wandoorensis* Hayat, 2015. To date, 13 species are recognized within the *proclia* group.

Despite the majority of *Aphytis* species having been assigned to species groups, a number of species remain unplaced, including several taxa considered “allied” or morphologically similar to existing groups. Currently, six species have been recorded as allied to the *proclia* group. Rosen and DeBach (1979) [9] reported five such species: *A. griseus* Quednau, 1964; *A. tucumani* Rosen & DeBach, 1979; *A. acrenulatus* DeBach & Rosen, 1976; *A. amazonensis* Rosen & DeBach, 1979; and *A. desantisi* DeBach & Rosen, 1976. Subsequently, Rosen and DeBach (1986) added *A. bangalorensis* Rosen & DeBach, 1986 [12]. These allied species resemble members of the *proclia* group morphologically but lack several of its key diagnostic features; for example, they exhibit weaker body pigmentation, reduced occipital stripes, and an absence of dark markings along the genal sutures.

Species of the *proclia* group are distributed worldwide, with their primary diversity concentrated in the Palaearctic and Oriental regions [9]. In China, seven species of the *proclia* group and two allied species have been recorded to date [13,14,15,16,17,18]. In this study, we collected four species of *Aphytis* from Fuzhou, Fujian Province, including one member of the *proclia* group and three species allied to this group. Here, we summarize the host associations and distribution records of these four species, provide detailed morphological descriptions and illustrations for the two new species, and reconstruct maximum likelihood phylogenetic trees based on partial COI and 28S rDNA sequences to elucidate their phylogenetic relationships. The molecular data not only support the morphological delimitation of the new species but also provide crucial evidence for reassessing their placement within or outside the *proclia* group. Furthermore, we present an updated checklist and a morphological key for the 11 Chinese species of the *proclia* group and its morphologically similar species. This study represents the first integrated taxonomic and molecular phylogenetic treatment of Chinese members of the *proclia* group and its allied species and highlights the necessity of combining morphological and molecular evidence for accurate species group assignment in *Aphytis*.

## 2. Materials and Methods

### 2.1. Collection and Rearing of Parasitoids

From 2024 to 2025, *Pinnaspis aspidistrae* (Signoret) and *Pinnaspis theae* (Maskell) were collected on plants of *Liriope* sp. and *Camellia* sp. in Fujian; we observed the species and dissected the parasitized scale insects in the laboratory. The larvae and pupae of *Aphytis* parasitoids obtained from scale insects were individually transferred to pre-numbered round plastic boxes (3 cm in diameter, 1.5 cm in height), in which filter paper was placed and half a drop of water was added daily to maintain humidity. When the pupae had developed to maturity (compound eyes turning from black to gray-green), we recorded their morphological characteristics and photographed them. They were reared until *Aphytis* parasitoids emerged as adults.

### 2.2. Morphological Examination

The body color of specimens was photographed using a Nikon DS-Ri2 camera (Nikon, Tokyo, Japan) with NIS-Elements Dv4.40 software, attached to a Nikon SMZ18 microscope (Nikon, Tokyo, Japan), and then the specimens were preserved in alcohol. Specimens subjected to non-destructive DNA extraction were placed in clean water to photograph their dorsal morphological features, including the scutellum, propodeum and abdominal tergites. Afterwards, specimens were dehydrated and slide-mounted for species identification following the method outlined by Noyes (1982) [19]. Slide-mounted specimens were photographed using a Nikon Ni microscope (Nikon, Tokyo, Japan) with the same camera and software, including forewings, antennae, head, and body. The body length of specimens was measured before they were slide-mounted, and all other measurements were completed on the slide-mounted specimens. Type material and the other specimens examined in the study were deposited in the College of Plant Protection, Fujian Agriculture and Forestry University, Fuzhou, Fujian, China (FAFU).

Identification of the species refers to the principles and methods in “Species of *Aphytis* of the world (Hymenoptera: Aphelinidae)” published by Rosen and DeBach (1979) [9]. The following abbreviations are used: F1, F2, etc. = antennal funicle segments 1, 2, etc.; T1, T2, etc. = gastral tergites 1, 2, etc.

### 2.3. Molecular Identification

Genomic DNA was extracted from *Aphytis* specimens using the DNeasy Blood & Tissue Kit (Qiagen, Düsseldorf, Germany) based on the non-destructive method proposed by Polaszek et al. (2013) [20]. Using the extracted DNA as a template, COI and 28S rDNA were amplified. PCR amplification was performed in a final volume of 25 µL containing 12.5 µL 2× Taq PCR MasterMix II (Sangon Biotech, Shanghai, China), 1 µL forward primer, 1 µL reverse primer, 2 µL genomic DNA and 8.5 µL ddH_2_O. The PCR primers and reaction conditions are shown in Table 1 and Table 2. The quality of the amplifications was verified via 1% agarose gel electrophoresis (Table 1 and Table 2). Sequencing primers were the same as the PCR primers, and the PCR products were sequenced at Sangon Biotech (Shanghai, China). The sequences were edited and verified using Geneious prime version 2023 comparing forward and reverse sequences. The sequences were submitted to the GenBank. Additional sequences for the phylogenetic analysis were download from NCBI (Table 3 and Table 4). All sequences were aligned using MAFFT v7.505 [21]. The best fit model was calculated using ModelFinder v2.2.0 [22]. The aligned sequences of COI and 28S rDNA were used to infer a maximum likelihood phylogenetic tree with IQ-TREE v2.2.0 [23] under the MGF3×4+G4 model and TIM3e + G4 model for 5000 ultrafast bootstraps. All phylogenetic analyses of sequences were conducted using Phylosiuit v1.2.3 software [24].

## 3. Results

### 3.1. Morphological Identification

#### 3.1.1. A Checklist of the *proclia* Group and Its Morphologically Similar Species from China

A checklist of the *proclia* group and its morphologically similar species from China is provided below. In total, eleven species are recognized, including seven species assigned to the *proclia* group and four morphologically similar species (two known species and two new species). Among them, *A. hispanicus*, *A. pinnaspidis*, *A. latarcuatus*, sp. n., and *A. denticulatus*, sp. n. were collected in this study; the remaining species were recorded from the literature.

***proclia* group (7 species):**

(1) *Aphytis comperei* DeBach & Rosen, 1976

Host: *Aonidiella aurantii*, *Parlatoria pergandii*, *Chrysomphalus aonidum*, *Mytilaspis gloverii*, and *Lepidosaphes beckii* (all on citrus) [9,16].

Distribution: China (Fujian, Guangdong, Hong Kong, Guangxi, Sichuan); the USA, Mexico, Jamaica, South Africa [9,16].

(2) *Aphytis confusus* DeBach & Rosen, 1976

Host: *Coccus longulus* on *Jasminum sambac*, *Ledaspis distincta* on *Protea caffra,* and *Africaspis chionaspiformis* or *Melanaspis corticos* [9,14].

Distribution: China (Taiwan); South Africa [9,14].

(3) Aphytis diaspidis (Howard, 1881)

Host: *Aonidiella aurantii* (on citrus), *Lepidosaphes gloverii* (on citrus), *Aspidiotus hederae*, *Diaspidiotus crataegivorus*, *D. perniciosus*, *Hemiberlesia juglandis*, *Diaspis echinocacti*, *D. bromeliae*, *Chrysomphalus aonidum*, *C. bifasciculatus*, *Aulacaspis rosae*, *Pseudaulacaspis pentagona*, *Lepidosaphes ulmi*, *Pinnaspis buxi*, and *Fiorinia fioriniae* [9,16].

Distribution: China (Fujian, Taiwan, Guangdong, Sichuan); Pakistan, Lebanon, Cyprus, Iran, Israel, Greece, Italy, France, the USA, Bermuda, Australia, New Zealand, South Africa, Eritrea, Argentina, Brazil, Peru, Chile, Mexico [9,14,16].

(4) *Aphytis* hispanicus (Mercet, 1912)

Host: *Parlatoria pergandii*, *P. cinerea*, *P. oleae*, *Chrysomphalus dictyospermi* on citrus, *Acutaspis scutiformis*, *Aspidiotus nerii*, *Lopholeucaspis japonica*, *Mytilaspis conchiformis* (=*Lepidosaphes ficus*), and *Insulaspis pallida* [9,13].

Distribution: China (Hunan, Fujian: Shaowu, Changle, Shaxian, Zhangzhou, Nanjing; Taiwan, Guangdong); the former USSR, Israel, Turkey, Italy, Spain, the USA, Trinidad, Jamaica, Morocco, Brazil, Mexico [9,13].

(5) *Aphytis* proclia (Walker, 1839)

Host: *Pseudaulacaspis pentagona* (on *Prunus salicina* and *Ligustrum* sp.), *Lepidosaphes salicina*, *Aulacaspis rosarum*, *Quadraspidiotus zonatus* (on *Quercus robur*), *Q. perniciosus*, *Q. forbesi* (on *Pyrus americana*), *Chionaspis salicis* (on *Tilia* sp. and *Salix* sp.), *C. salicisnigrae* (on *Salix* sp.), *C. americana* (on *Ulmus americana*), *Lepidosaphes ulmi* (on *Syringa vulgaris*), *Diaspidiotus* sp. (on *Pyrus* sp.), *Chrysomphalus dictyospermi* (on *citrus* sp. and *rosa* sp.), *Aonidiella aurantii*, *Hemiberlesia lataniae* (reared host), and *Aspidiotus nerii* (reared host) [9,13,27].

Distribution: China (Heilongjiang: Harbin; Liaoning: Pulandian, Haicheng; Shaanxi, Zhejiang, Jiangxi, Hunan, Fujian: Fuzhou, Yongtai; Taiwan, Guangdong, Sichuan); the former USSR, Japan, Myanmar, Cyprus, the UK, France, Germany, Italy, Austria, Hungary, the USA, El Salvador, North Africa, Mexico [9,13].

(6) *Aphytis testaceus* Chumakova, 1961

Host: *Diaspidiotus ostreaeformis, Epidiaspis leperii*, *Lepidosaphes ulmi*, *L. salicina*, *L. granati*, and *Carulaspis minima* [9,28].

Distribution: China (Heilongjiang: Harbin); Russia, Azerbaijan, Hungary, Moldova [9,17,28].

(7) *Aphytis vandenboschi* DeBach & Rosen, 1976

Host: *Quadraspidiotus perniciosus* (on pine) and *Hemiberlesia pitysophila* (on pine) [9,18].

Distribution: China (Guangdong: Panyu, Shenzhen, Doumen, Taishan, Xinhui, Boluo, Huidong); Japan [9,18].

**Morphologically similar species (4 species):**

(1) *Aphytis bangalorensis* Rosen et & DeBach, 1986

Host: one species of armored scale insect; *Pseudaulacaspis rubra* (=*P. barberi*) on mango [12,15];

Distribution: China: Yunnan (Kunming); India [12,15].

(2) *Aphytis pinnaspidis* Rosen & DeBach, 1979

Host: *Pinnaspis aspidistrae* (Signoret) on *Liriope* sp., *P. strachani* (Cooley), *P*. nr. *strachani*, *Aonidiella aurantii* (Maskell) [9].

Distribution: China (Fujian: Fuzhou); Brazil; Mexico; the USA [9].

(3) *Aphytis latarcuatus* Wang & Huang, sp. n.

Host: *Pinnaspis theae* (Maskell) on *Camellia* sp.

Distribution: China (Fujian: Fuzhou).

(4) *Aphytis denticulatus* Wang & Huang, sp. n.

Host: *Pinnaspis aspidistrae* (Signoret) on *Liriope* sp.

Distribution: China (Fujian: Fuzhou).

#### 3.1.2. Key to the Chinese Species of the *proclia* Group and Its Morphologically Similar Species

The following key is based on morphological characters and covers seven species of the *proclia* group and four morphologically similar species known from China.

**Key to species of the *proclia* group and allied species of *Aphytis* from China**

1. Distinct black bars on the occiput and genal sutures; body coloration more or less dusky, with fuscous markings; thoracic sterna conspicuously pigmented; forewings distinctly clouded......................................................................................*proclia* group......2
  Head lacks the distinct pigmentation feature; body coloration pale yellow to gray, with infuscated or fuscous markings; thoracic sterna dusky; forewings without distinctly clouded.....................................................allied species of *proclia* group...............8
2. Abdominal tergites with complete transverse fuscous crossband...............................3
  Abdominal tergites with incomplete short transverse fuscous stripes, but T3 and T7 with a complete crossband...................................................................................................4
3. Antennal pedicel, funicular and club uniformly dusky; propodeal crenulae 6+7–7+8, rounded, low and wide; abdominal tergites with rather faint, transverse fuscous crossband; ovipositor long, 1.89–2.13 times as long as the middle tibia.........*A. testaceus*
  Antennal funicular and club fuscous, apex of club dusky; propodeal crenulae 3+3–6+7, rounded, more elongate than *A. testaceus*; abdominal tergites with conspicuously transverse fuscous crossband; ovipositor 1.33–1.75 times as long as the middle tibia.........................................................................................................................*A. diaspidis*
4. Both T3 and T7 with a complete transverse fuscous crossband......................................5
  T3 or T7 without a complete transverse fuscous crossband............................................6
5. Antennal pedicel, funicular and base of club uniformly infuscated, apex of club dusky; propodeal crenulae 3+4–8+8, rather rounded; ovipositor 1.33–2.00 times as long as the middle tibia; uniparental..........................................................*A. vandenboschi*
  Antennal pedicel and funicular fuscous, base of club paler than funicular, apex of club dusky; propodeal crenulae 5+5–10+10, elongate; ovipositor 1.41–1.61 times as long as the middle tibia; biparental.......................................................................*A. proclia*
6. Apex of antennal club gradually narrow; propodeal crenulae elongate, nonoverlapping; only T3 with a complete transverse fuscous crossband; uniparental............................................................................................................................................7
  Antennal club tapering strongly toward a pointed apex; propodeal crenulae 5+5–6+7, distinctly rounded, not elongate, closely appressed, slightly overlapping; each side of T3 with short transverse fuscous stripes; biparental.................................*A. confusus*
7. Antennal club 2.50–3.00 times as long as wide, apical third of club blackish; propodeal crenulae faintly infuscated, 6+6–8+8, rather narrow; ovipositor 1.46–1.71 times as long as the middle tibia; forewings relatively narrow..............................*A. hispanicus*
  Antennal club 2.20–2.40 times as long as wide, tip of club with conspicuously black spot; propodeal crenulae dusky, 3+4–5+5, somewhat wide, ovipositor 1.69–2.00 times as long as the middle tibia; forewings relatively broad...................................*A. comperei*
8. Body without distinct markings; propodeum very long, longer than 1.15 times the length of the scutellum.........................................................................................................9
  Body with infuscated markings; propodeum relatively long, 0.80–1.10 times as long as the scutellum...................................................................................................................10
9. The posterior margin of propodeum with a rather prominent median projection; crenulae rounded, 4+3–4+4, closely appressed, forming a continuous row on the projection, nonoverlapping; uniparental..........................................*A. denticulatus*, sp. n.
  The posterior margin of propodeum relatively protruding, broadly arcuate; crenulae slightly pointed, 6+5–6+6; biparental..................................................*A. latarcuatus*, sp. n.
10. Body yellow; propodeum 0.88–1.06 times as long as the scutellum; crenulae 3+3–5+5, large, somewhat elongate and overlapping; ovipositor long, 1.80–2.20 times as long as the middle tibia................................................................................*A. bangalorensis*
  Body grayish yellow; propodeum 1.02–1.11 times as long as the scutellum; crenulae minute or *absent*, not elongate, nonoverlapping; ovipositor short, 1.35–1.53 times as long as the middle tibia....................................................................................*A. pinnaspidis*

#### 3.1.3. *Aphytis hispanicus* (Mercet, 1912) (Figure 1)

Host: *Pinnaspis aspidistrae* (Signoret) on *Liriope* sp., *Parlatoria pergandii* Comstock, *Chrysomphalus dictyospermi* (Morgan), *Parlatoria oleae* (Colvee), *Aspidiotus nerii* (Bouche) [9,13].

Distribution: China (Hunan, Fujian: Shaowu, Changle, Shaxian, Zhangzhou, Nanjing; Taiwan, Guangdong); the former USSR, Israel, Turkey, Italy, Spain, the USA, Trinidad, Jamaica, Morocco, Brazil, Mexico [9,13].

Material examined: 1♀1♂, China: Fujian, Fuzhou, Jinshan, FAFU, 14.iii.2025, ex. *Pinnaspis aspidistrae* (Signoret) on *Liriope* sp. coll. Jingtao Xi.

Note. *Aphytis hispanicus* is a uniparental species, with conspicuous black bars on the occiput and genal sutures [9]. Body coloration more dusky, with fuscous markings on the body and appendages. The species belongs to the *proclia* group and resembles *A. proclia* (Walker), differing from the latter in the mode of reproduction and in certain details of body coloration, antennae and forewings.

**Figure 1:**
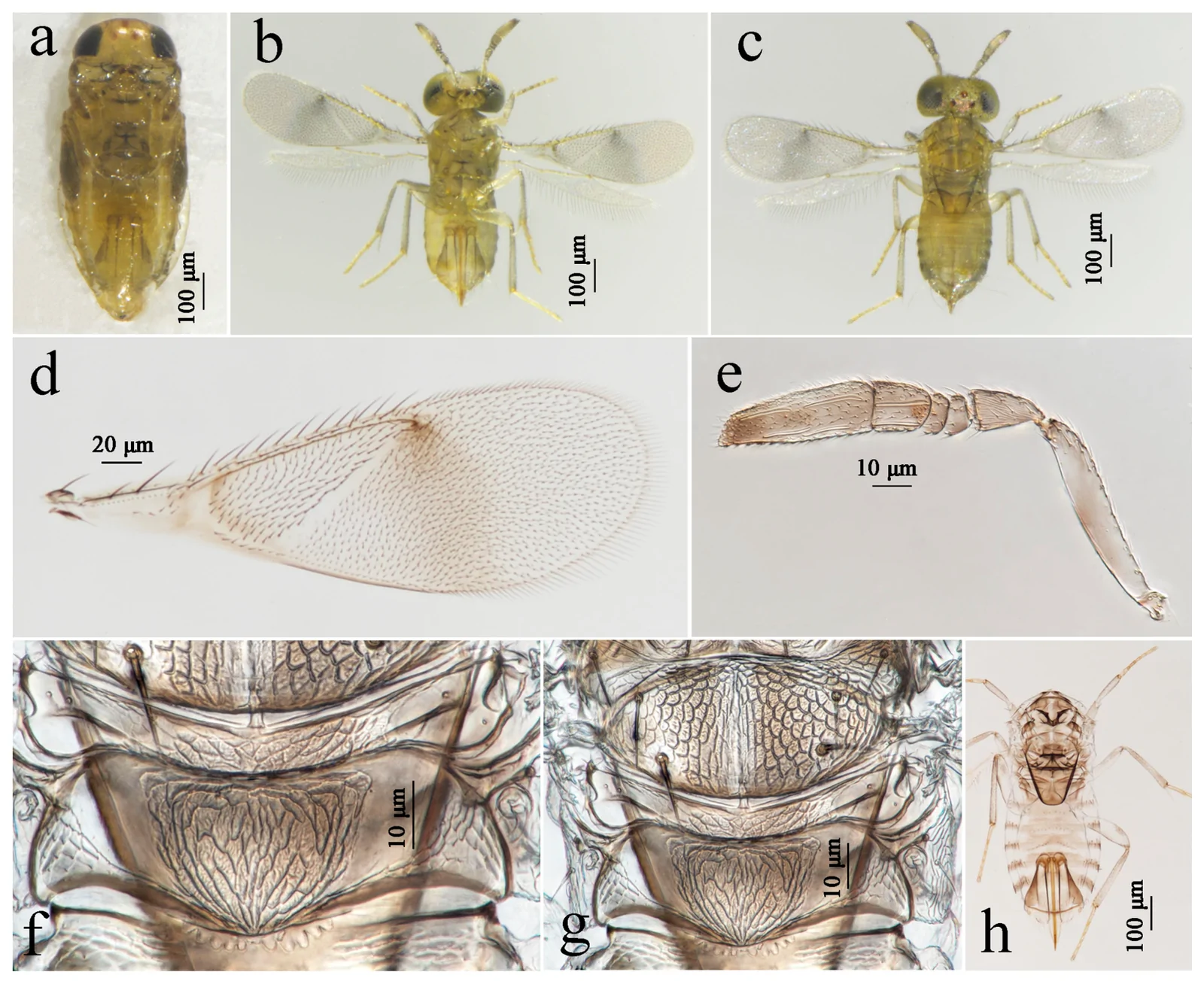
*Aphytis hispanicus* (♀). (**a**) Pupa in ventral view; (**b**) adult in ventral view; (**c**) adult in dorsal view; (**d**) forewing; (**e**) antenna; (**f**) propodeum; (**g**) scutellum and propodeum; (**h**) mesosoma and metasoma in ventral view (showing mid-tibia and ovipositor).

#### 3.1.4. *Aphytis pinnaspidis* Rosen & DeBach, 1979 (Figure 2)

Host: *Pinnaspis aspidistrae* (Signoret) on *Liriope* sp., *P. strachani* (Cooley), *P*. nr. *strachani*, *Aonidiella aurantii* (Maskell) [9].

Distribution: China (Fujian), Brazil, Mexico, America [9].

Material examined: 5♀, China: Fujian, Fuzhou, Jinshan, FAFU, 25.iii.2024, ex. *Pinnaspis aspidistrae* (Signoret) on *Liriope* sp. coll. Jingtao Xi.

Note. *Aphytis pinnaspidis* is regarded as a member of the *proclia* group [9]. As found in this study, the species has blackish bars on the occiput, but genal sutures without conspicuous markings and pigmentation, as in *A. latarcuatus*, sp. n. and *A. denticulatus*, sp. n.; so, *A. pinnaspidis* may not be an actual member of the *proclia* group, but rather a related species of the group.

**Figure 2:**
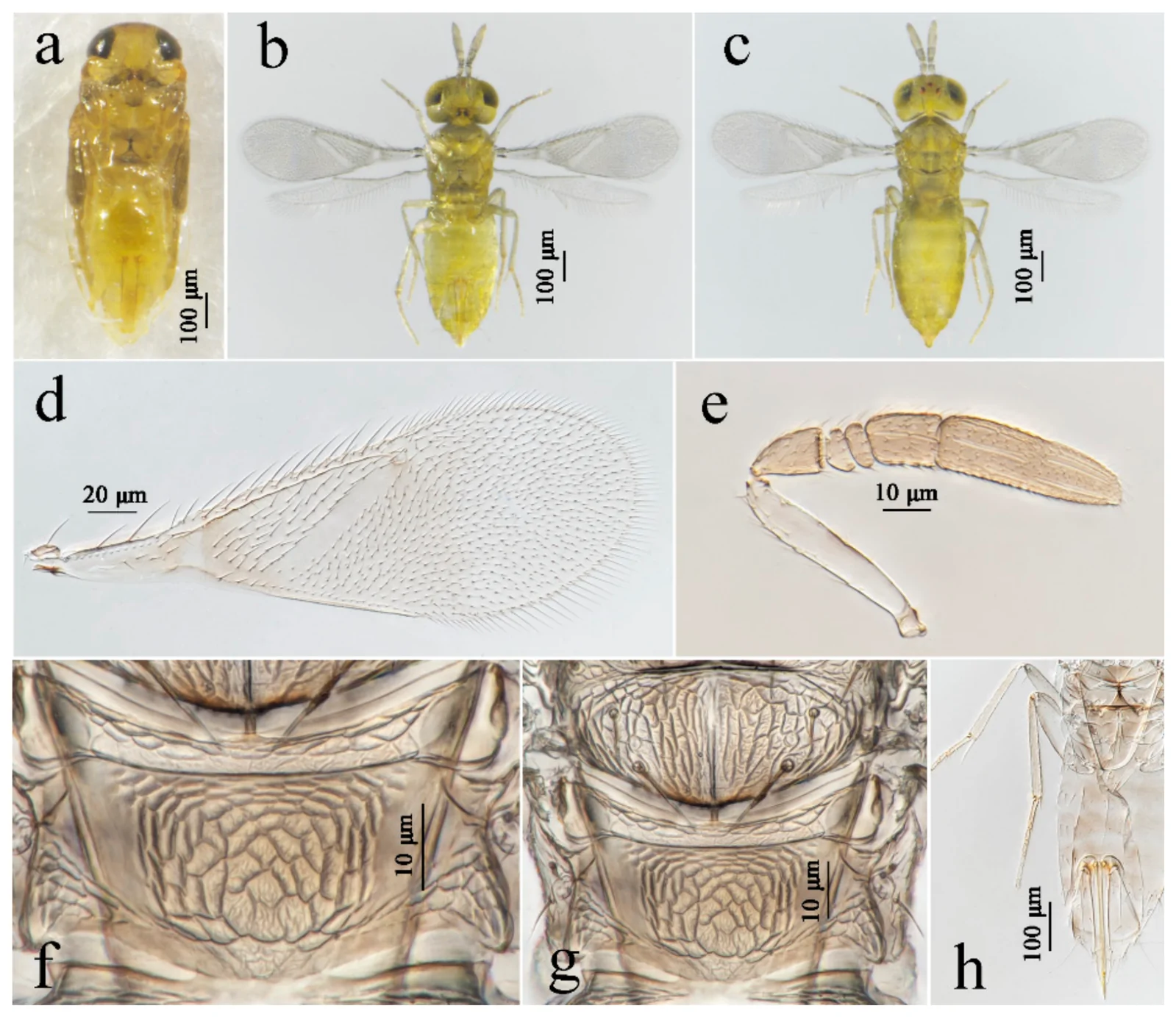
*Aphytis pinnaspidis* Rosen & DeBach (♀). (**a**) Pupa in ventral view; (**b**) adult in ventral view; (**c**) adult in dorsal view; (**d**) forewing; (**e**) antenna; (**f**) propodeum; (**g**) scutellum and propodeum; (**h**) part of mesosoma and metasoma in ventral view (showing mid-tibia and ovipositor).

#### 3.1.5. *Aphytis denticulatus* Wang & Huang, sp. n. (Figure 3 and Figure 4)

*Diagnosis*. *Aphytis denticulatus*, sp. n. is a uniparental species, usually coexisting with *A. hispanicus* and *A. pinnaspidis* in the same habitat. The new species resembles *A. pinnaspidis* in the grayish-yellow coloration of its body with slight markings, but can be distinguished from the latter by the long propodeum (usually longer than 1.20 times the length of the scutellum) with a rather prominent median projection and rounded crenulae (*A. pinnaspidis*: propodeum 1.00–1.10 times as long as the scutellum; crenulae minute or absent). The new species lacks the characteristic cephalic pigmentation and may be related to, but not an actual member of, the *proclia* group.

*Description*. **Female.** Body length 0.78–0.87 mm. **Colour.** Body grayish-yellow with slight markings and brown mandibles; faintly infuscated bars on the occiput; genal sutures immaculate. Pronotum and anterior margin of mid-lobe of mesoscutum faintly dusky; scutellum faintly dusky and posterior margin of scutellum narrowly lined with black; propodeum and each side of petiole faintly dusky. A black spot on each side of prothoracic sternum and dusky transversal stripe on the central part of prothoracic sternum; mesosternum faintly dusky with the longitudinal stem of the furca (“Y”) marked with a blackish color. Ventral side of abdomen yellow. Antenna and legs yellowish. Forewing hyaline without conspicuously clouded spot. **Head.** Eyes finely setose. Mandibles with two denticles and a dorsal truncation. Antenna with six segments, and antennal formula 1,1,3,1. Scape slender, varying from 5.23 to 5.92 times as long as wide, longer than club; pedicel 1.54–1.58 times as long as wide, about as long as F3; F1 trapezoidal as wide as F2; F2 symmetrical, 0.45–0.48 times as long as wide; F3 rectangular, 1.14–1.18 times as long as wide, bearing two longitudinal sensilla; club 3.10–3.40 times as long as wide, 2.83–2.92 times as long as F3, bearing six longitudinal sensilla. **Mesosoma.** Mid-lobe of mesoscutum with 10 setae; each side lobe of mesoscutum with 2 setae; each axilla with 1 seta; scutellum with 4 setae, the posterior pair conspicuously longer than the anterior pair, the placoid sensilla usually closer to the posterior than to the anterior pair of setae. Propodeum conspicuously longer than the scutellum, 1.21–1.29 times as long as the scutellum, with a rather prominent median projection, reticulate on the sides and central area, the cells wide and somewhat rectangular; crenulae rounded, 4+3–4+4, closely appressed, forming a continuous row on the projection, nonoverlapping. **Forewing.** 2.62–2.76 times as long as wide; marginal fringe not exceeding 0.20 times width of disk; delta area with 40–45 setae in 4–5 rows, longer and sparser than the setae in lateral of the linea calva; submarginal vein bearing 2 coarse and long setae; marginal vein bearing 9–11 subequal and prominent setae along anterior margin. **Leg.** Mid-tibial spur usually about as long as the corresponding basitarsus; tarsal formula 5-5-5. **Metasoma.** Petiole transversely reticulate on both sides, smooth centrally; TI–TVI reticulate on the sides, bearing 2 fine setae in a transverse row on each reticulate area; TI–TV faintly transversely striated anteriorly across center, longitudinally striated posteriorly; TVI reticulate centrally; TV–TVI with 2 setae centrally; TVII triangular, bearing 6 setae in a row. Ovipositor short, located centrally at TIII, 1.35–1.39 times as long as the middle tibia.

Male. Unknown.

Pupa. The mature pupa of *A. denticulatus*, sp. n. is entirely yellow, wing pads slightly brown; thoracic sterna faintly dusky, with a black spot on each side of the prothoracic sternum and a longitudinal black line on the mesosternum.

Host. *Pinnaspis aspidistrae* (Signoret) on *Liriope* sp.

Distribution. China (Fujian).

Etymology. The new species was named after the gear-shaped crenulae.

Material. Holotype ♀, China: Fujian, Fuzhou, Jinshan, FAFU, 10.v.2025, ex. *Pinnaspis aspidistrae* (Signoret) on *Liriope* sp. coll. Jingtao Xi (FAFU); Paratypes 2♀, same data as holotype (FAFU).

**Figure 3:**
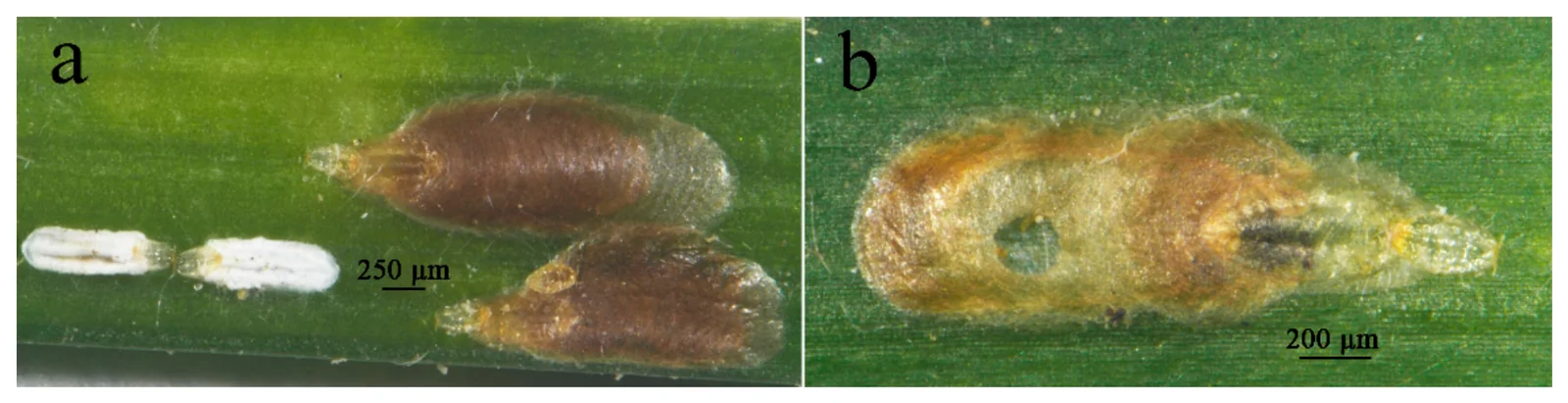
Host and emergence holes of *Aphytis denticulatus*, sp. n. (**a**) *Pinnaspis aspidistrae* on *Liriope* sp.; (**b**) emergence holes on host scale.

**Figure 4:**
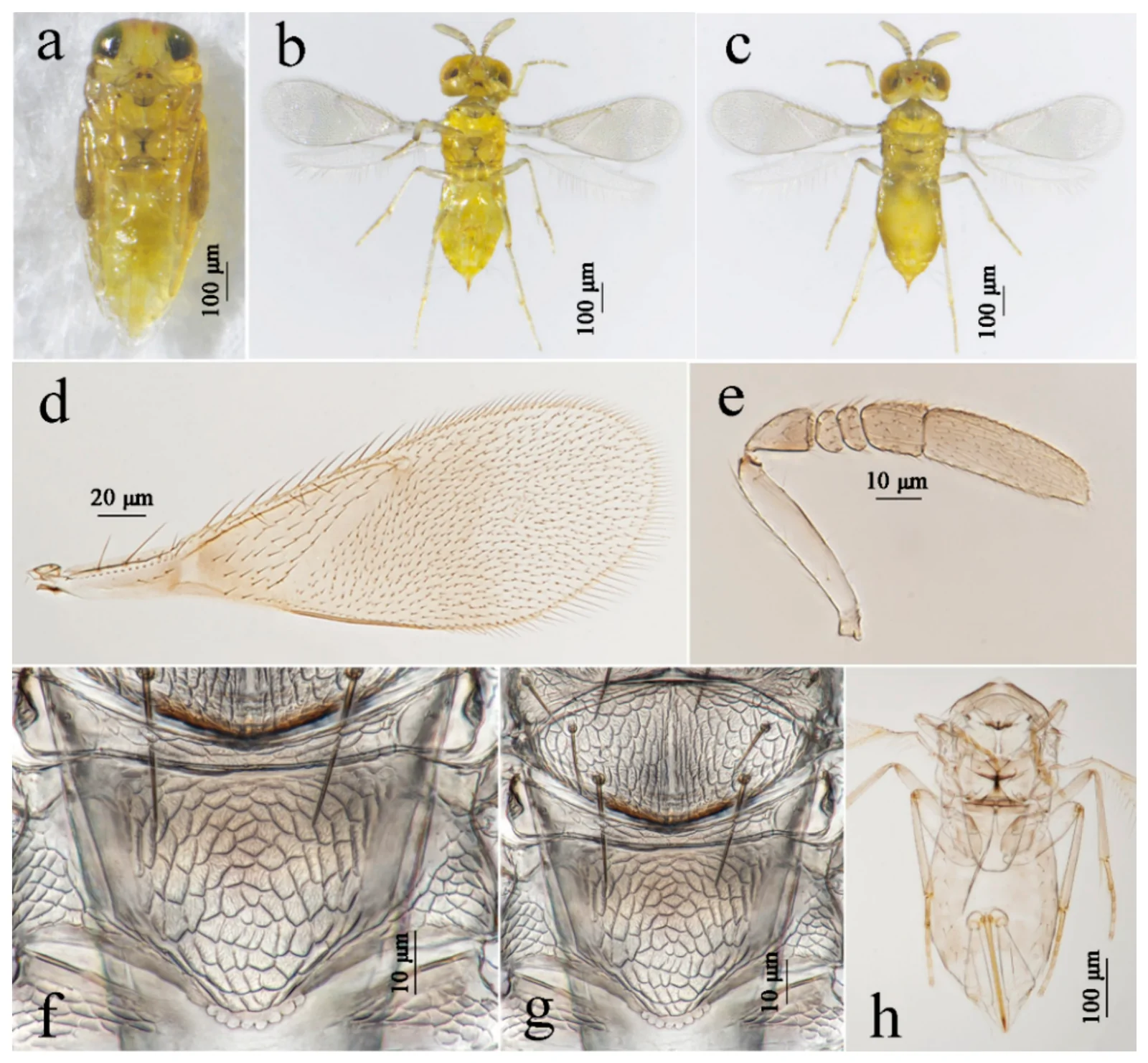
*Aphytis denticulatus*, sp. n. (Holotype, ♀). (**a**) Pupa in ventral view; (**b**) adult in ventral view; (**c**) adult in dorsal view; (**d**) forewing; (**e**) antenna; (**f**) propodeum; (**g**) scutellum and propodeum; (**h**) mesosoma and metasoma in ventral view (showing mid-tibia and ovipositor).

#### 3.1.6. *Aphytis latarcuatus* Wang & Huang, sp. n. (Figure 5, Figure 6 and Figure 7)

*Diagnosis.* This biparental species is very closely related to *A. denticulatus*, sp. n. in the grayish-yellow coloration of its body with little markings and the absence of characteristic cephalic pigmentation, differing from the latter in the mode of reproduction and in certain details of structure. *A. latarcuatus*, sp. n. can be recognized by the following combination of characteristics: the posterior margin of the long propodeum is relatively protruding, not as prominent as *A. denticulatus*, sp. n.; crenulae 5+5–5+6, small, slightly pointed (*A. denticulatus*, sp. n.: crenulae rounded, 4+3–4+4, closely appressed); F3 somewhat more slender than *A. denticulatus*, sp. n.; forewing slightly narrower and longer than *A. denticulatus*, sp. n. Like *A. denticulatus*, sp. n., *A. latarcuatus*, sp. n. is related to, but not a member of, the *proclia* group.

*Description*. **Female.** Body length 0.72–0.84 mm. **Colour.** General coloration similar to that of *A. denticulatus*, sp. n., grayish-yellow body; a pair of faintly infuscated bars on the occiput; genal sutures without markings and pigmentation. Pronotum fuscous; scutellum faintly infuscated, the posterior margin lined with a blackish color; propodeum and sides of petiole more strongly infuscated. A black spot on each side of prothoracic sternum, infuscated transversal stripe on the central part of the prothoracic sternum; mesosternum with a conspicuous longitudinal median black line on the stem of the mesosternal furca (“Y”). Legs and antenna pale yellow. Forewing hyaline, a short black streak at the base of the forewing. **Head.** Eyes finely setose. Mandibles as in *A. denticulatus*, sp. n. Antenna six segments; scape slender, 5.37–5.50 times as long as wide, as long as or somewhat longer than club; pedicel 1.61–1.89 times as long as wide, nearly as long as F3; F1 slightly trapezoidal, 0.69–0.72 times as long as wide; F2 more symmetrical, slightly shorter, 0.40 times as long as wide; F3 1.33–1.50 times as long as wide, bearing 1–2 longitudinal sensillum; club 3.07–3.20 times as long as wide, 2.67–2.75 times as long as F3, bearing 5–6 longitudinal sensilla. **Mesosoma.** Mid-lobe of mesoscutum with 10–11 setae; each parapsis with 2 setae; each axilla with 1 seta; scutellum with 4 setae, the posterior pair setae longer, the discoid sensilla somewhat closer to the posterior than to the anterior pair. Propodeum long, 1.18–1.27 times as long as the scutellum, reticulate on the sides and on a broad central area; posterior margin relatively protruding, broadly arcuate, but without a distinct median salient; crenulae small, 5+5–5+6, slightly pointed, forming a continuous row along median salient of posterior margin, nonoverlapping. **Forewing.** 2.93–3.15 times as long as wide, somewhat narrower and longer than *A. denticulatus*, sp. n.; marginal fringe not exceeding 0.20 times width of disk; delta area with 40–47 setae in 5–6 rows, longer and sparser than the setae in lateral of linea calva; submarginal vein bearing 2 coarse setae; marginal vein bearing 7–10 prominent, subequal setae along anterior margin. **Leg.** Mid-tibial spur nearly as long as the corresponding basitarsus; tarsal formula 5-5-5. **Metasoma.** Similar to *A. denticulatus*, sp. n. in the sculpture and chaetotaxis of abdominal tergites. Petiole transversely reticulate on both sides, central area without a reticulate; TI–V transversely striated centrally, a reticulate on both sides, a row of 2–3 setae on each side of the reticulate area; TV–VI with 2 setae centrally; TVII with 6–7 setae in a row. Ovipositor short, located centrally at TIII, 1.27–1.32 times as long as the middle tibia.

**Male**. Body length 0.66–0.75 mm. Similar to the female in structure, chaetotaxis, sculpture and coloration. Antennal scape wider than that of the female, 4.60 times as long as wide, longer than the club; pedicel 1.60 times as long as wide; F3 1.60 times as long as wide, bearing 1–2 longitudinal sensillum; club 3.36 times as long as wide, 2.31 times as long asF3, bearing 2–3 longitudinal sensilla. Thorax, propodeum and forewings as in the female. Genitalia 0.60 times as long as the middle tibia.

**Pupa.** The mature pupa of *A. latarcuatus*, sp. n. is entirely yellow, similar to *A. denticulatus*, sp. n. in pigmentation.

*Host. Pinnaspis theae* (Maskell) on *Camellia* sp.

*Distribution.* China (Fujian).

*Etymology.* The new species was named after the shape of the propodeum posterior margin, as broadly arcuate.

Material. Holotype♀, China: Fujian, Fuzhou, Jinshan, FAFU, 21.v.2024, ex. *Pinnaspis theae* (Maskell) on *Camellia* sp. coll. Jingtao Xi (FAFU). Paratypes 1♀1♂, same data as holotype (FAFU).

**Figure 5:**
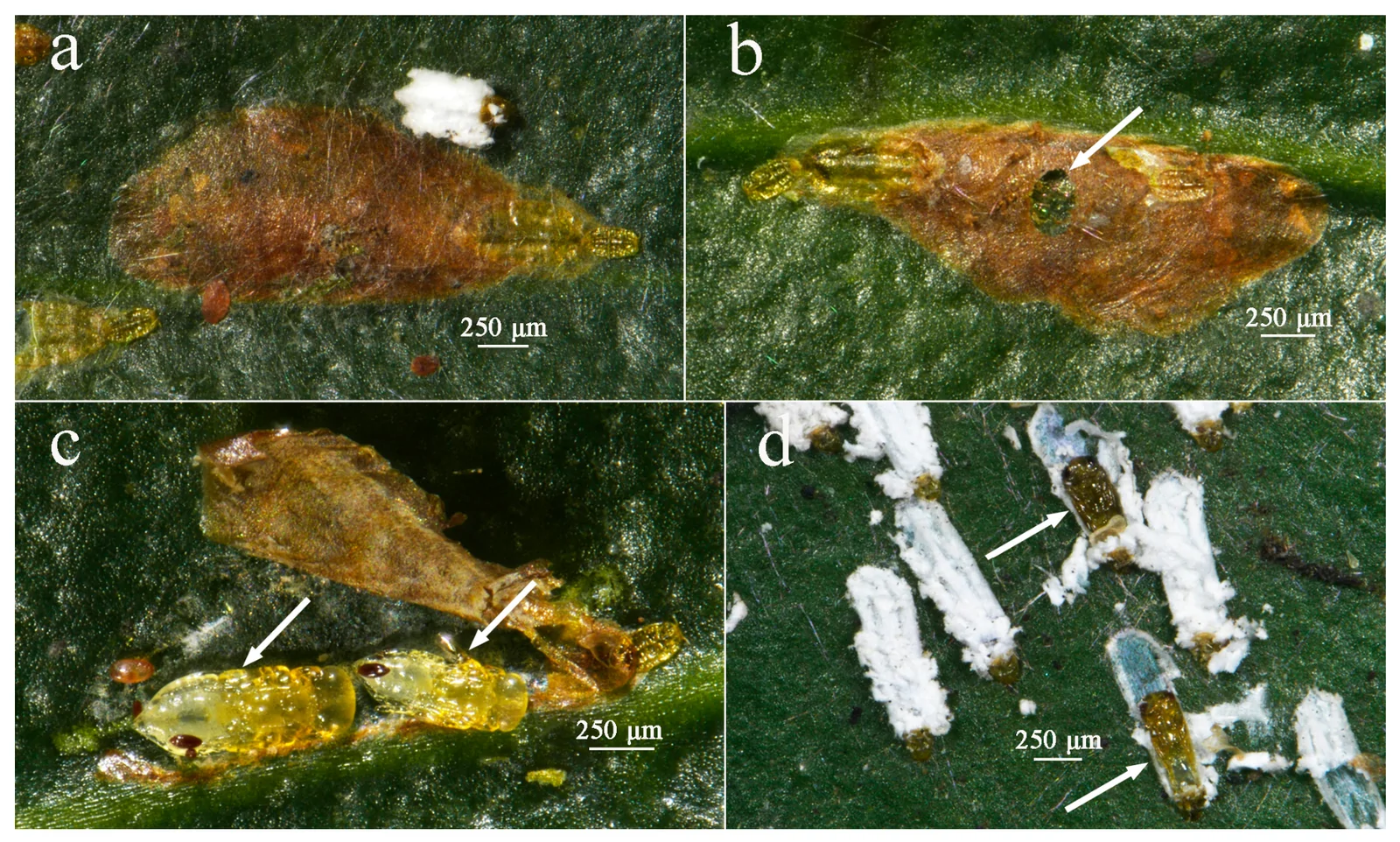
Host, emergence holes and pupae of *Aphytis latarcuatus*, sp. n. (**a**) *Pinnaspis theae* on *Camellia* sp.; (**b**) emergence hole; (**c**) pupae on female scales; (**d**) pupae on male scales.

**Figure 6:**
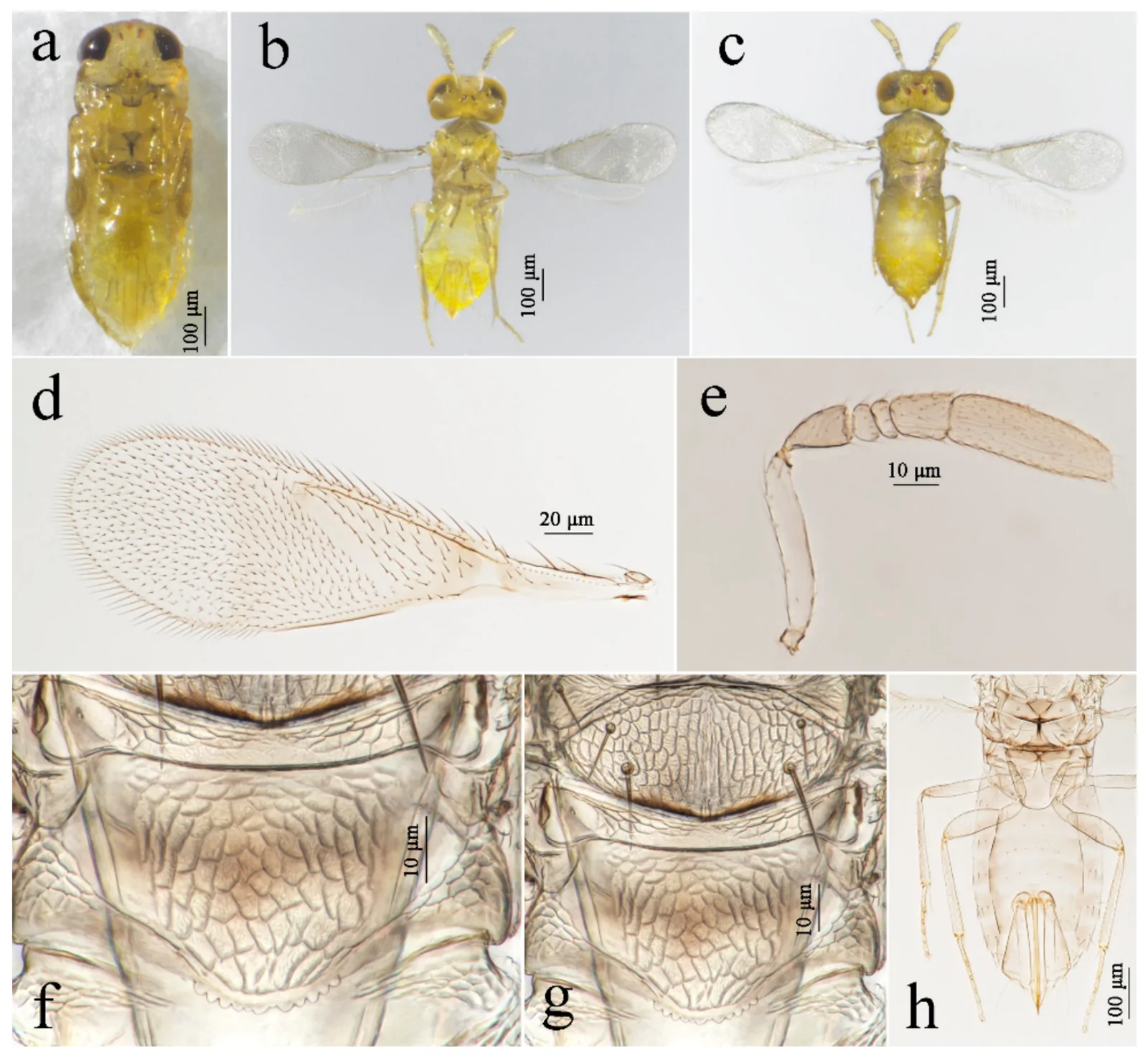
*Aphytis latarcuatus*, sp. n. (Holotype, ♀). (**a**) Pupa in ventral view; (**b**) adult in ventral view; (**c**) adult in dorsal view; (**d**) forewing; (**e**) antenna; (**f**) propodeum; (**g**) scutellum and propodeum; (**h**) part of mesosoma and metasoma in ventral view (showing mid-tibia and ovipositor).

**Figure 7:**
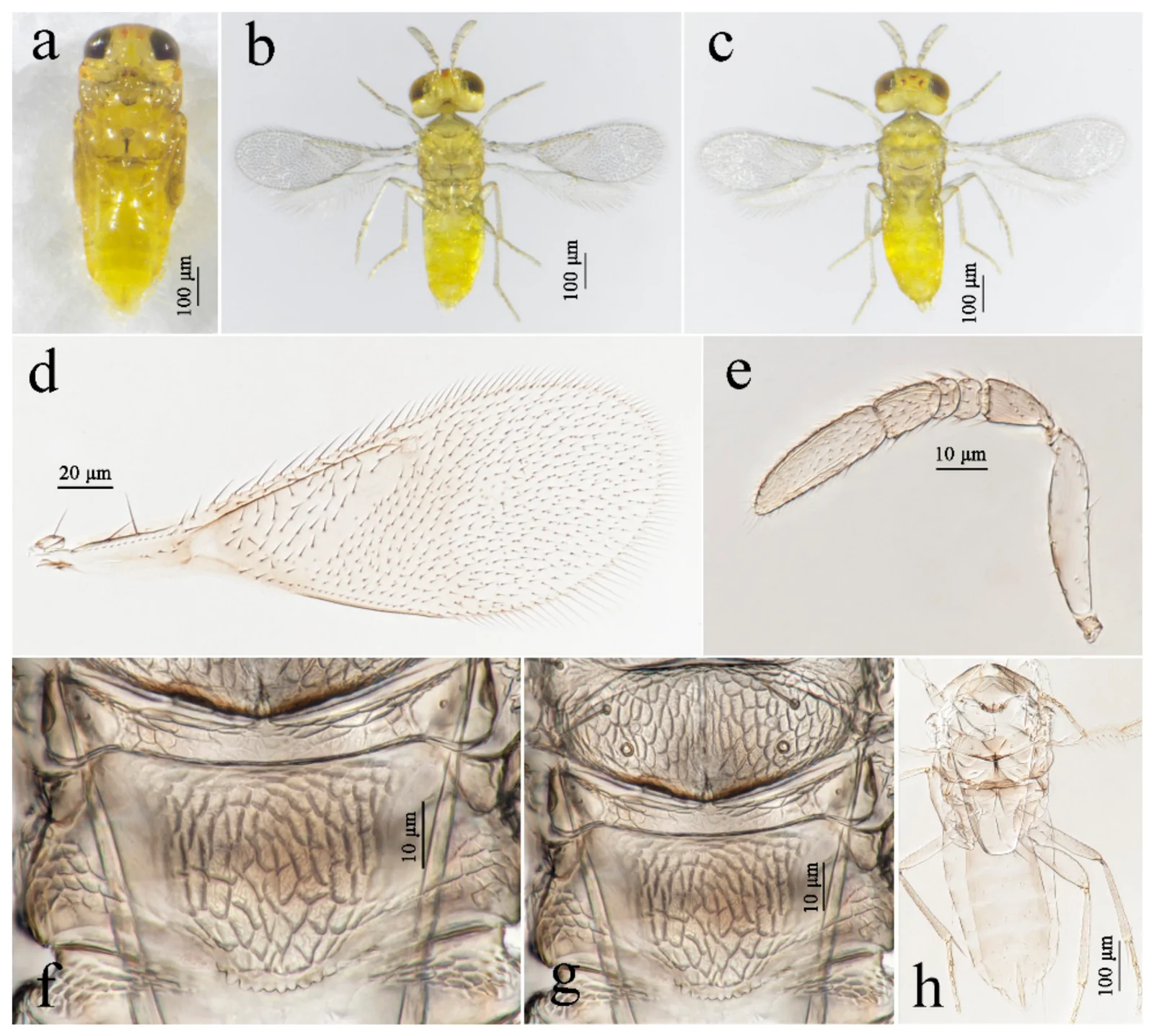
*Aphytis latarcuatus*, sp. n. (Paratype, ♂). (**a**) Pupa in ventral view; (**b**) adult in ventral view; (**c**) adult in dorsal view; (**d**) forewing; (**e**) antenna; (**f**) propodeum; (**g**) scutellum and propodeum; (**h**) mesosoma and metasoma in ventral view (showing mid-tibia and genitalia).

### 3.2. Phylogenetic Analysis

**Figure 8:**
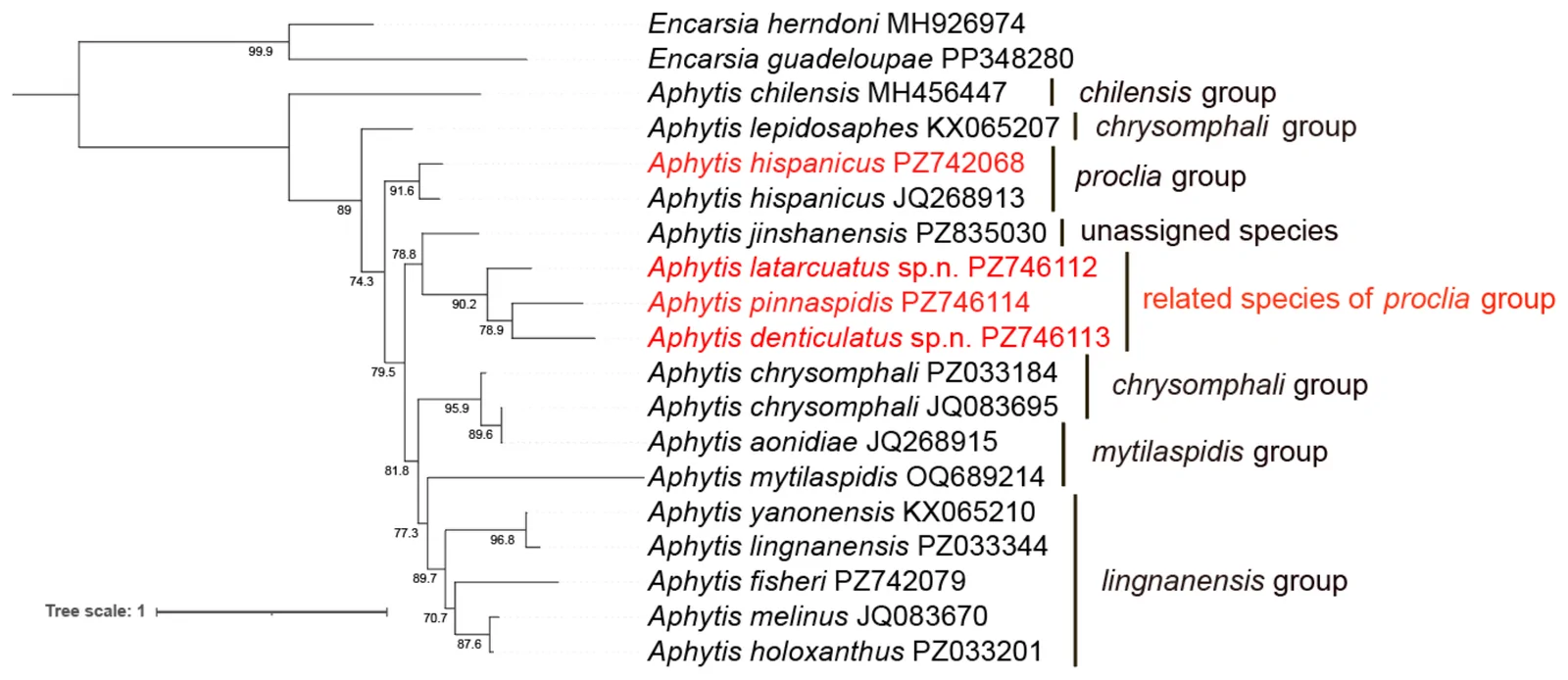
ML phylogenetic tree based on COI sequences (support values > 50% are shown at nodes), note: scientific names highlighted in red indicate the species of this paper.

**Figure 9:**
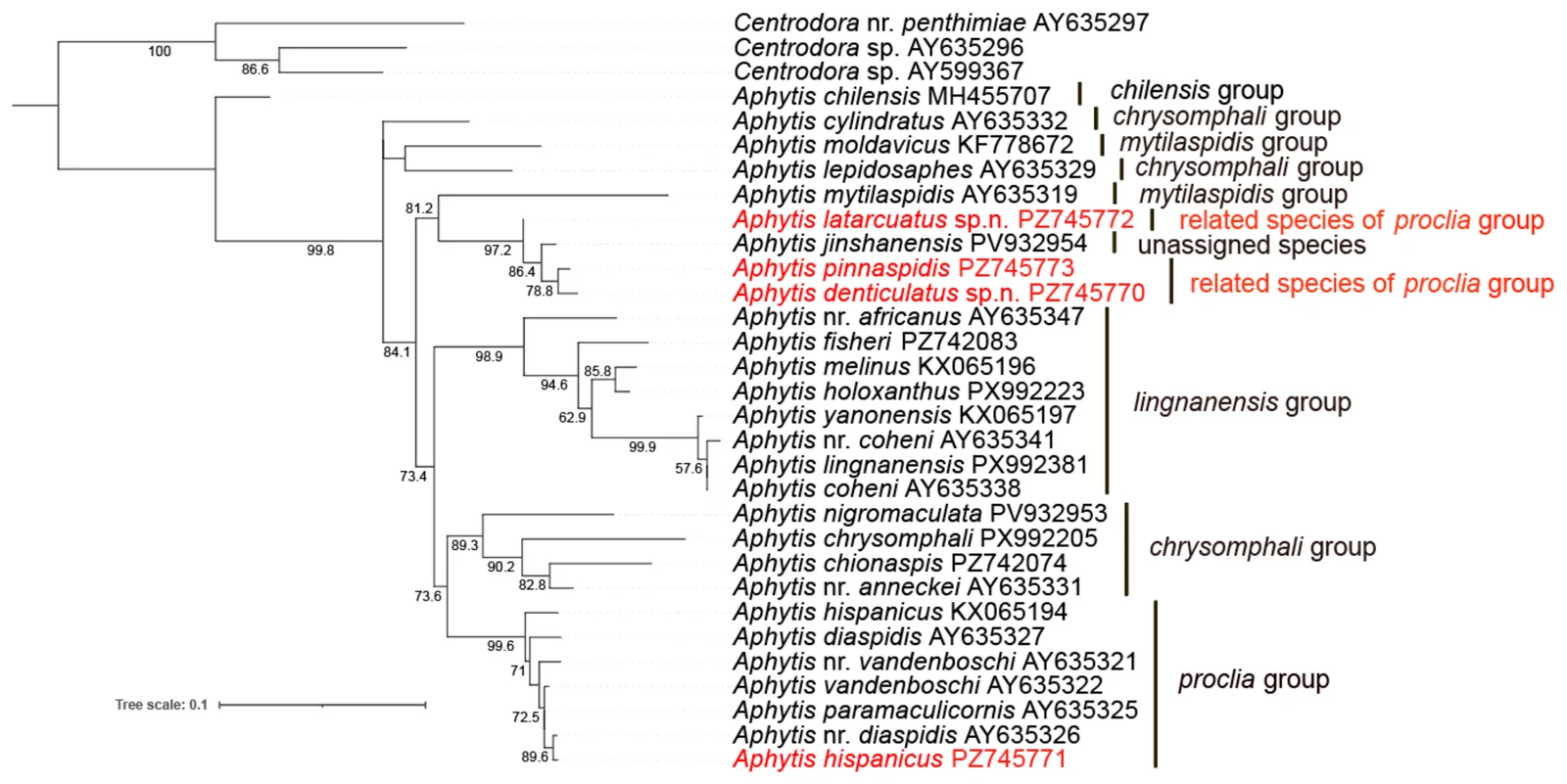
ML phylogenetic tree based on 28S rDNA sequences (support values > 50% are shown at nodes), note: scientific names highlighted in red indicate the species of this paper.

In the maximum likelihood tree (based on COI), *A. pinnaspidis*, *A. denticulatus*, sp. n. and *A. latarcuatus*, sp. n. clustered on a branch (90.2% support values), in which *A. pinnaspidis* and *A. denticulatus*, sp. n. formed a sister group. *A. hispanicus* is an independent branch, clearly separated from *A. pinnaspidis*, *A. denticulatus*, sp. n. and *A. latarcuatus*, sp. n. (Figure 8). Their molecular phylogenetic relationships are consistent with the results of morphological identification. In morphology, *A. hispanicus* belongs to the *proclia* group. *A. pinnaspidis*, *A. denticulatus*, sp. n. and *A. latarcuatus*, sp. n. are related to the *proclia* group, but are not actual members of the *proclia* group. In addition, *A. jinshanensis* is placed in a branch (78.8% support values) with related species of the *proclia* group (Figure 8). In morphology, *A. jinshanensis* belongs to the unassigned species, but the species have some important morphological characteristics similar to *A. pinnaspidis*, *A. denticulatus*, sp. n. and *A. latarcuatus*, sp. n. The maximum likelihood tree based on 28S rDNA is similar to the former. All species of the *proclia* group cluster on a large branch (99.6% support values), formed a monophyletic group. *A. pinnaspidis*, *A. denticulatus*, sp. n. and *A. latarcuatus*, sp. n. cluster on the other branch and are far away from the clade of the *proclia* group. The results also support that the above three *Aphytis* species do not belong to the *proclia* group. In addition, *A. jinshanensis* is placed in a branch (97.2% support values) with related species of the *proclia* group (Figure 9).

## 4. Discussion

In this study, *Aphytis denticulatus*, sp. n. and *A. latarcuatus,* sp. n. are treated as morphologically similar species (allied species) to the *proclia* group. Both morphological and molecular data consistently support their exclusion from this group. In the 28S rDNA tree, the two new species are placed outside the *proclia* group clade, suggesting that their morphological resemblance to the group may result from plesiomorphic retention rather than close phylogenetic affinity.

The taxonomic placement of *A. pinnaspidis* also requires revision. Rosen and DeBach (1979) [9] assigned this species to the *proclia* group based on overall morphological similarity. However, our detailed morphological re-examination reveals that *A. pinnaspidis* does not fully conform to the diagnostic features of the *proclia* group, although it is morphologically similar to members of this group and should be treated as an allied species. According to Rosen and DeBach (1979) [9], the key distinction between members of the *proclia* group and their allied species lies in the pigmentation pattern and dark markings on the head: members of the *proclia* group possess conspicuous dark markings on both the occipital margin and the genal sutures, whereas allied species have pigmentation only on the occipital margin or genal sutures. Rosen and DeBach (1979) [9] described the genal sutures of *A. pinnaspidis* as pale brown, but our examination confirms that the pigmentation on the genal sutures of *A. pinnaspidis* is indistinct and virtually indistinguishable from that observed in the two new species, while differing markedly from the strongly pigmented genal sutures of *A. hispanicus*, a typical member of the *proclia* group. Therefore, *A. pinnaspidis* should be excluded from the *proclia* group.

The molecular phylogenetic analyses based on COI and 28S rDNA sequences strongly support the morphological conclusions. In the COI tree, *A. pinnaspidis* and the two new species form a distinct clade with 90.2% bootstrap support. In the 28S rDNA tree, the *proclia* group is recovered as a strongly supported monophyletic clade (99.6% bootstrap), while *A. pinnaspidis* and the two new species form an independent clade (97.2% bootstrap) that is completely excluded from the *proclia* group.

In the COI tree, *A. jinshanensis* is placed in a branch (78.8% support values) with *A. pinnaspidis*, *A. denticulatus*, sp. n., and *A. latarcuatus*, sp. n. In the 28S rDNA tree, *A. jinshanensis* is placed within the same clade (97.2% bootstrap support) together with *A. pinnaspidis*, *A. denticulatus*, sp. n., and *A. latarcuatus*, sp. n. This species was previously considered unassigned within *Aphytis*, with no clear affinity to any known species groups. Our results reveal a close relationship between *A. jinshanensis* and these three species, suggesting that this clade may represent a previously unrecognized species group lineage within *Aphytis*. Morphologically, these four species share several key characters: (1) a relatively long propodeum, equal to or longer than the scutellum; (2) a black spot on each side of the prothoracic sternum; and (3) relatively pale body coloration. Among these, the presence of black spots on the prothoracic sternum has not been emphasized in the existing literature but appears consistently in this lineage, suggesting that it may serve as a useful diagnostic characteristic for recognizing this potential new lineage.

The results of this study indicate that head pigmentation patterns, long considered a key diagnostic feature of the *proclia* group, may be more variable than previously recognized and may not be reliable as the sole basis for delimiting the group. This highlights the necessity of integrating morphological and molecular data in species group-level taxonomy.

This study presents the first molecular phylogenetic analysis of Chinese species of the *proclia* group and its allied species, but several limitations should be acknowledged. The molecular analyses included only four of the eleven Chinese species recognized in this study (*A. hispanicus*, *A. pinnaspidis*, and the two new species) plus *A. jinshanensis* from GenBank. The remaining seven species (*A. testaceus*, *A. diaspidis*, *A. comperei*, *A. confusus*, *A. proclia*, *A. vandenboschi*, and *A. bangalorensis*) lack molecular data. Consequently, the full membership of this potential new lineage remains uncertain and awaits further verification with additional molecular data.

In conclusion, this study clarifies the taxonomic status of several species previously associated with the *proclia* group in China, describes two new species, and provides the first molecular evidence for the existence of a distinct lineage within *Aphytis*. The results underscore the importance of integrating morphological and molecular data for accurate species group assignment and provide a foundation for future studies on the diversity, phylogeny, and biological control potential of *Aphytis* in China and beyond.

## Figures and Tables

**Table 1:** Primers and cycling conditions (COI) [25].

Primer	Sequence	Cycling Conditions			
LCO1490F	5′-GGTCAACAAATCATAAAGATATTGG-3′	Denaturation	Annealing	Extension	Cycles
HCO2198R	5′-TAAACTTCAGGGTGACCAAAAAATCA-3′	94 °C (30 s)	50 °C (90 s)	72 °C (60 s)	35

**Table 2:** Primers and cycling conditions (28S rDNA) [26].

Primer	Sequence	Cycling Conditions			
D2-3551F	5′-CGTGTTGCTTGATAGTGCAGC-3′	Denaturation	Annealing	Extension	Cycles
D2-4068R	5′-TTGGTCCGTGTTTCAAGACGGG-3′	94 °C (30 s)	58 °C (90 s)	72 °C (60 s)	35

**Table 3:** The species and GenBank accession numbers used in phylogenetic analysis (COI).

Genus/Species	GenBank Accession	Source
Genus *Aphytis* Howard, 1900		
*Aphytis aonidiae* (Mercet, 1911)	JQ268915	GenBank
*Aphytis chilensis* Howard, 1900	MH456447	GenBank
*Aphytis chrysomphali* (Mercet, 1912)	JQ083695	GenBank
*Aphytis chrysomphali* (Mercet, 1912)	PZ033184	GenBank
***Aphytis denticulatus*** **sp. n.**	PZ746113	This paper
*Aphytis fisheri* DeBach, 1959	PZ742079	GenBank
***Aphytis hispanicus*** **(Mercet, 1912)**	PZ742068	This paper
*Aphytis hispanicus* (Mercet, 1912)	JQ268913	GenBank
*Aphytis holoxanthus* DeBach, 1960	PZ033201	GenBank
*Aphytis jinshanensis* Wang & Huang, 2025	PZ835030	GenBank
***Aphytis latarcuatus*** **sp. n.**	PZ746112	This paper
*Aphytis lepidosaphes* Compere, 1955	KX065207	GenBank
*Aphytis lingnanensis* Compere, 1955	PZ033344	GenBank
*Aphytis melinus* DeBach, 1959	JQ083670	GenBank
*Aphytis mytilaspidis* (Le Baron, 1870)	OQ689214	GenBank
***Aphytis pinnaspidis*** **Rosen & DeBach, 1979**	PZ746114	This paper
*Aphytis yanonensis* DeBach & Rosen, 1982	KX065210	GenBank
Genus *Encarsia* Förster, 1878		
*Encarsia guadeloupae* Viggiani, 1987	PP348280	GenBank
*Encarsia herndoni* (Girault, 1935)	MH926974	GenBank

**Table 4:** The species and GenBank accession numbers used in phylogenetic analysis (28S rDNA).

Genus/Species	GenBank Accession	Source
Genus *Aphytis* Howard, 1900		
*Aphytis chilensis* Howard, 1900	MH455707	GenBank
*Aphytis chionaspis* Ren Hui, 1988	PZ742074	GenBank
*Aphytis chrysomphali* (Mercet, 1912)	PX992205	GenBank
*Aphytis coheni* DeBach, 1960	AY635338	GenBank
*Aphytis cylindratus* Compere, 1955	AY635332	GenBank
***Aphytis denticulatus*** **sp. n.**	PZ745770	This paper
*Aphytis diaspidis* (Howard, 1881)	AY635327	GenBank
*Aphytis fisheri* DeBach, 1959	PZ742083	GenBank
***Aphytis hispanicus*** **(Mercet, 1912)**	PZ745771	This paper
*Aphytis hispanicus* (Mercet, 1912)	KX065194	GenBank
*Aphytis holoxanthus* DeBach, 1960	PX992223	GenBank
*Aphytis jinshanensis* Wang & Huang, 2025	PV932954	GenBank
***Aphytis latarcuatus*** **sp. n.**	PZ745772	This paper
*Aphytis lepidosaphes* Compere, 1955	AY635329	GenBank
*Aphytis lingnanensis* Compere, 1955	PX992381	GenBank
*Aphytis melinus* DeBach, 1959	KX065196	GenBank
*Aphytis moldavicus* Yasnosh, 1966	KF778672	GenBank
*Aphytis mytilaspidis* (Le Baron, 1870)	AY635319	GenBank
*Aphytis nigromaculata* Wang & Huang, 2025	PV932953	GenBank
*Aphytis* nr. *africanus* Quednau, 1964	AY635347	GenBank
*Aphytis* nr. *anneckei* DeBach & Rosen, 1976	AY635331	GenBank
*Aphytis* nr. *coheni* DeBach, 1960	AY635341	GenBank
*Aphytis* nr. *diaspidis* (Howard, 1881)	AY635326	GenBank
*Aphytis* nr. *vandenboschi* DeBach & Rosen, 1976	AY635321	GenBank
*Aphytis paramaculicornis* DeBach & Rosen, 1976	AY635325	GenBank
***Aphytis pinnaspidis*** **Rosen & DeBach, 1979**	PZ745773	This paper
*Aphytis vandenboschi* DeBach & Rosen, 1976	AY635322	GenBank
*Aphytis yanonensis* DeBach & Rosen, 1982	KX065197	GenBank
Genus *Centrodora* Förster, 1878		
*Centrodora* nr. *penthimiae* Annecke, 1965	AY635297	GenBank
*Centrodora* sp.	AY599367	GenBank
*Centrodora* sp.	AY635296	GenBank

## Data Availability

Data are contained within the article.
